# An efficient algorithm for protein structure comparison using elastic shape analysis

**DOI:** 10.1186/s13015-016-0089-1

**Published:** 2016-09-29

**Authors:** S. Srivastava, S. B. Lal, D. C. Mishra, U. B. Angadi, K. K. Chaturvedi, S. N. Rai, A. Rai

**Affiliations:** 1 ICAR-Indian Agricultural Statistics Research Institute, New Delhi, India; 2 Biostatistics Shared Facility, James Graham Brown Cancer Center, University of Louisville, Louisville, USA; 3 Department of Bioinformatics and Biostatistics, University of Louisville, Louisville, USA; 4 Centre for Agricultural Bioinformatics, ICAR-Indian Agricultural Statistics Research Institute, Library Avenue, New Delhi, 110012 India

**Keywords:** Protein structure comparison, Backbone atoms, Geodesic distance, Side chain properties

## Abstract

**Background:**

Protein structure comparison play important role in in silico functional prediction of a new protein. It is also used for understanding the evolutionary relationships among proteins. A variety of methods have been proposed in literature for comparing protein structures but they have their own limitations in terms of accuracy and complexity with respect to computational time and space. There is a need to improve the computational complexity in comparison/alignment of proteins through incorporation of important biological and structural properties in the existing techniques.

**Results:**

An efficient algorithm has been developed for comparing protein structures using elastic shape analysis in which the sequence of 3D coordinates atoms of protein structures supplemented by additional auxiliary information from side-chain properties are incorporated. The protein structure is represented by a special function called square-root velocity function. Furthermore, singular value decomposition and dynamic programming have been employed for optimal rotation and optimal matching of the proteins, respectively. Also, geodesic distance has been calculated and used as the dissimilarity score between two protein structures. The performance of the developed algorithm is tested and found to be more efficient, i.e., running time reduced by 80–90 % without compromising accuracy of comparison when compared with the existing methods. Source codes for different functions have been developed in R. Also, user friendly web-based application called ProtSComp has been developed using above algorithm for comparing protein 3D structures and is accessible free.

**Conclusions:**

The methodology and algorithm developed in this study is taking considerably less computational time without loss of accuracy (Table 2). The proposed algorithm is considering different criteria of representing protein structures using 3D coordinates of atoms and inclusion of residue wise molecular properties as auxiliary information.

## Background

Comparison of protein structures is an important for understanding structural, functional and evolutionary relationship among protein specially in case of novel proteins [1]. In addition to this, it is being extensively used for identifying homologous residues [2, 3], finding recurrent folds [4], identifying structural motifs and functional sites, searching similar structure in structural database, predicting interaction among residues/proteins, and hierarchical classification of proteins [5–10]. Structural analysis of proteins is much more important than sequence analysis as protein structures are more conserved than sequences [1, 11]. The comparison of protein can also be used for evaluation of sequence alignment methods [12, 13], prediction of unknown protein structures and evaluation of predicted 3D structure of a protein.

In the last two decades, research in the area of protein structure comparison has gained momentum but the problem of finding optimal alignment having significant role in biological context still continues [1]. Number of methods for comparing two protein structures has been proposed in the literature. These methods are either based on various distance measures or scoring schemes. There is strong need to develop standard scoring function [14, 15] based on strong theoretical foundation as majority of existing techniques are heuristic in nature [1]. These existing techniques are not only less accurate but have more computational time and space complexity [16]. Hence, there is a scope for improvement in the existing methods for better comparison of protein structures [1, 15, 17].

Algorithms of two protein 3D structures comparison approaches can be broadly classified into two categories, i.e., (1) is based on rigid body alignment by super positioning protein structures heuristically with scaling, rotation, transformation and then super-positioning [18] and (2) based on fragmentation of structures and assembling by non-sequential alignment [18, 19]. The techniques of first category can perform better when the protein structures are small and each having equal number of residues in their sequences. The basic limitations of second category are selection of appropriate fragments size, computational time and space complexity for alignments. Various metrics for comparing and scoring identity between two protein structures are employed in both category of approaches, but the most commonly used are p values and root mean square deviation (RMSD). These metrics are rarely used for protein structure comparison with respect to single technique. Further, method such as Distance mAtrix aLIgnment (DALI) employ similarity score which is not a metric but it uses heuristic rule to search the neighborhoods based on strong matches [20]. Comparing of these techniques with respect to implementation and their practical utilities, these methods are difficult to use practically due to space and time complexity [21].

Recently, an attempt has been made for protein structure comparison using geodesic distance as dissimilarity score based on a particular Riemannian metric [22]. In this technique 3D coordinates of backbone atoms have been used to derive parameterized curve in real numbers in three dimensional space i.e. R^3^, for representing the protein structures. The alignment of two protein structures is being defined as the alignment of the two curves derived from backbone atoms of two structures i.e., one from each protein. Each of these parameterized curve is represented by a special function called square root velocity function (SRVF). Further, shapes comparison has been done after removing all shape preserving transformations from these curves. It has been pointed out that this comparison can be improved further by using higher dimensional composite curves by concatenating the geometric (3D) coordinates with primary and secondary structures as auxiliary coordinates [23, 24] and side chain atoms. These side chain atoms play an important role in determination of protein structure and consequently protein functions. The orientations of side chains and molecular properties of residues have significant effect on protein conformational dynamics and hence the protein function [25]. Therefore, the inclusion of the side chain atoms and molecular properties are likely to improve this protein structures comparative analysis and it may lead to a better alignment as compared to the alignment obtained from existing techniques.

Therefore, in this study an attempt has been made to develop a method/algorithm based on the elastic shape analysis [26–29] considering both geometrical and molecular properties of protein. In the proposed algorithm, side chain atoms along with molecular properties such as hydrophobicity, polarity, orientation (dihedral angles), mass of residues, functional group type (aliphatic, acyclic, hydroxyl or sulphur-containing, aromatic) and number of side-chain atoms as auxiliary information have been included. The proposed technique requires significantly less time without compromising with the accuracy for comparing protein structures. The developed algorithm has been implemented using open source R software. The method has been elaborated stepwise in the “Proposed algorithm” section. The performance of the developed method was compared with the existing methods i.e., ESA [22, 23], combinatorial extension (CE) [30] and jFATCAT [31], Matt [32], multiple structural alignment algorithm (MUSTANG) [33] for which the details are provided in the “Results and discussion” section. Our method was found to be more accurate for classification purpose and efficient in terms of computational time.

## Proposed algorithm

The concept of shape elastic metric has been employed for calculating deformation and quantifying the difference between two 3D structures of proteins. This concept of shape and shape metric was developed by Kendall [34] for quantification and modelling of shapes. This includes analysis of shapes, detecting and tracking patterns in the images, classification and clustering of images, finding trajectory and path of objects, morphological changes in objects, etc. Further, it has been observed that SRVF and elastic metric performed better in comparison to their counterparts during its applications in many fields such as image analysis, movies analysis, RNA and protein 3D structure comparison etc. [22, 35]. Hence, in this study, SRVF and shape elastic metric have been employed for comparing proteins 3D structures.

An algorithm for comparison of two protein 3D structures based on elastic shape analysis [22, 34, 35] has been developed and implemented as web based tool for comparing two protein structures. This tool requires PDB files [36] as input and provides geodesic distance along with graphical display of optimal matching and superposed protein curves as an output for visualization.

### a. Algorithm

In the proposed algorithm, both geometric properties from 3D coordinates of atoms and molecular properties having significant role in protein folding were considered to derive a curve from protein structure (PDB file). Geometric properties are derived in three criteria from 3D coordinates of atoms for each residue of a protein, i.e., (1) by using the backbone (N, C_α_ and C) atoms (ESA-BB), (2) using C_α_ atoms only (ESA-CA) and (3) the mean coordinates of backbone atoms for each residue (ESA-MC-BB). Additionally, dihedral angles (phi, psi and omega) are included as compulsion in criteria (2) and (3). The molecular properties considered for development of this algorithm are hydrophobicity, polarity, mass of residues functional group type (aliphatic, acyclic, hydroxyl or sulphur-containing, aromatic) and number of side-chain atoms. These factors are used as auxiliary information [37–40]. In case of glycine, only the backbone atoms are being considered as an exception.

**Fig. 1:**
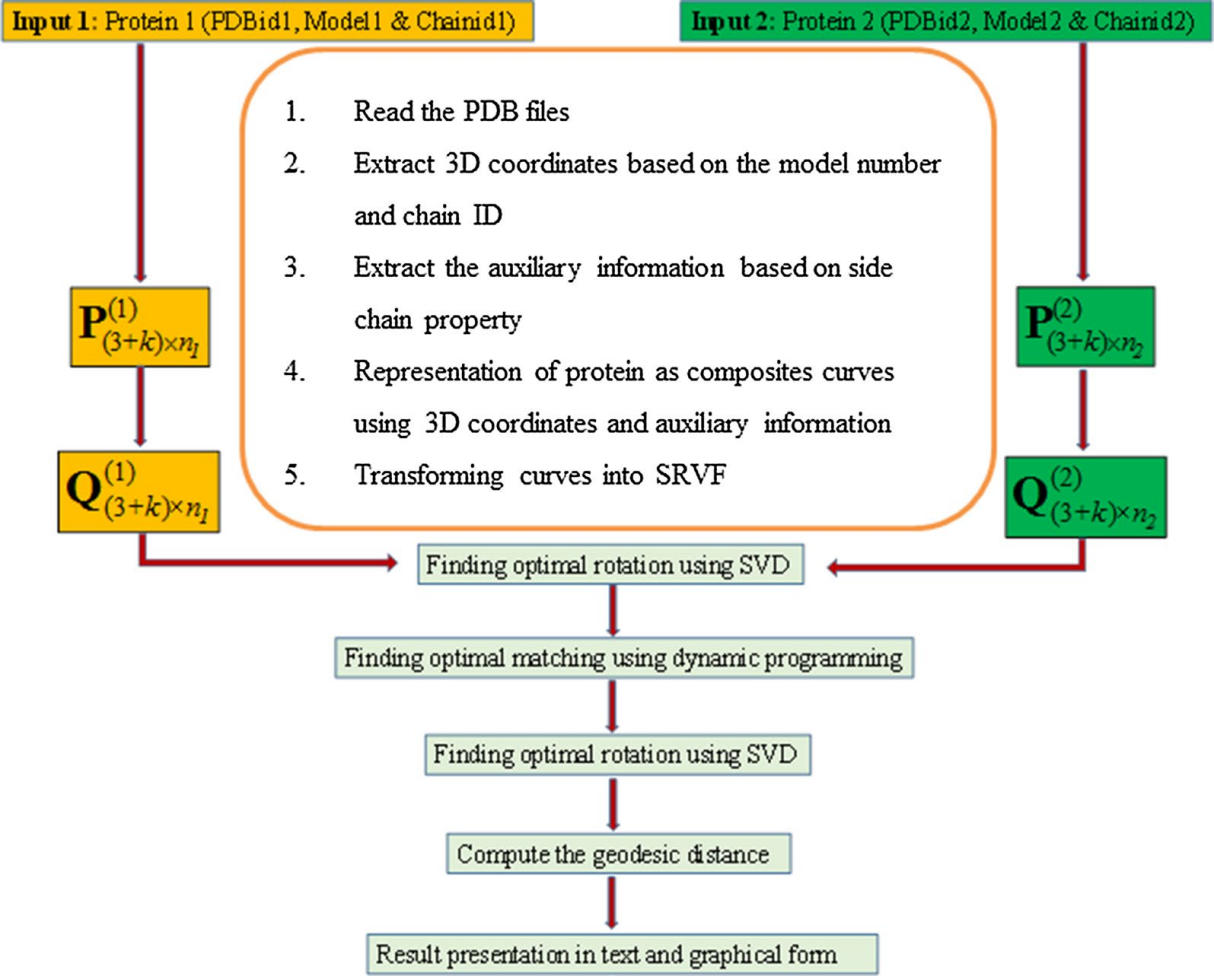
Flowchart of the algorithm

The steps involved in the proposed algorithm are given below and a flow chart represents the same as shown in Fig. 1.

#### Step 1

Extract 3D coordinates and auxiliary information to derive the initial input curve, ${\mathbf{P}}_{{\text{(3 + }k\text{)} \times n_{j} }}^{{\text{(}j\text{)}}}$ as given below, for each protein *j* (PDB File *j*) of length *n*_*j*_:

$${\mathbf{P}}_{{(3 + k) \times n_{j} }}^{(j)} = \left[ {\begin{array}{*{20}c} {p_{1,1}^{(j)} } & {p_{1,2}^{(j)} } & \ldots & {p_{{1,n_{j} }}^{(j)} } \\ {p_{2,1}^{(j)} } & {p_{2,2}^{(j)} } & \ldots & {p_{{2,n_{j} }}^{(j)} } \\ \vdots & \vdots & \ddots & \vdots \\ {p_{(3 + k),1}^{(j)} } & {p_{(3 + k),2}^{(j)} } & \ldots & {p_{{(3 + k),n_{j} }}^{(j)} } \\ \end{array} } \right]$$

Here, the superscript *j, j* = 1 and 2, refers to the protein 1 and protein 2, respectively. The subscript (3 + *k*) refers to the first 3 i.e. x, y, z coordinates of atoms and *k* coordinates are auxiliary information.

#### Step 2

Translate and scale by transforming the curves to their SRVFs, $Q_{{\left( {3 + k} \right)n_{j} }}^{(j)}$ corresponding to their piecewise linear function ${\rm T}^{j}$, respectively. This transformation for any given protein *j (j* = *1 or 2)* is as follows:

$$t_{1}^{(j)} = 0$$

$$t_{i + 1}^{(j)} = t_{i + 1}^{(j)} + \left| {\left| {\left( {p_{{1, \left( {i + 1} \right)}}^{\left( j \right)} , p_{{2, \left( {i + 1} \right)}}^{\left( j \right)} ,p_{{3, \left( {i + 1} \right)}}^{\left( j \right)} } \right) - \left( {p_{1, i}^{\left( j \right)} , p_{2, i}^{\left( j \right)} ,p_{3, i}^{\left( j \right)} } \right)} \right|} \right| \quad for\; i = 1,2, \ldots (n_{j} - 1)$$

$$T^{j } = \frac{1}{{t_{{n_{j} }}^{(j)} }} \left[ {t_{1}^{(j)} t_{2}^{(j)} \ldots t_{{n_{j} }}^{(j)} } \right] = \left[ {T_{1}^{(j)} T_{2}^{(j)} \ldots T_{{n_{j} }}^{(j)} } \right]$$

Therefore, first and last terms for both **T**^**1**^ and **T**^**2**^ are 0 and 1, and all the intermediate values will lie between 0 and 1.

$$Q_{{\left( {3 + k} \right)Xn_{j} }}^{(j)} = \frac{{\frac{{ dP_{{\left( {3 + k} \right)Xn_{j} }}^{(j)} }}{{dT^{(j)} }}}}{{\sqrt {\left\| {\frac{{ dP_{{\left( {3 + k} \right)Xn_{j} }}^{(j)} }}{{dT^{(j)} }}} \right\|} }}$$

#### Step 3

Recalculate the SRVFs $Q_{1}^{(1)} \;{\text{and}}\;Q_{1}^{(1)}$ corresponding to a new **T** (obtained by merging the unique values of parameter values) for each of dimension (3 + *k*) × *n*. Calculation is shown below:

$$\eqalign{ & {\text{T}} = {\text{unique}}\left[ {{{\text{T}}^1}{\text{ }}{{\text{T}}^2}} \right]{\mkern 1mu} \cr & \;\; = \left[ {0{\text{ }}T_2^{(1)}T_3^{(1)}T_4^{(1)}T_{{n_1} - 1}^{(1)} \ldots T_2^{\left( 2 \right)}T_3^{\left( 2 \right)}T_4^{\left( 2 \right)}T_{{n_2} - 1}^{\left( 2 \right)}} \right] \cr}$$

These values are arranged in increasing order and then the unique values are merged. It may be noted that the value of *n* will lie between max(*n*_*1*_, n_2_) and *n*_*1*_ + *n*_*2*_ − 2. The recalculated SRVFs, ${\bf{Q}}_{(3 + k) \times n}^{(1)}$ and ${\bf{Q}}_{(3 + k) \times n}^{(2)}$ corresponding to new **T** can be conveniently represented by **Q**_**1**_ and **Q**_**2**_ for protein 1 and protein 2, respectively.

#### Step 4

Obtain optimal rotation using SVD by following points given below

4.1 SVD (**A**) = **USV**^**T**^, where **A** = **Q**_**1**_**Q**_**2**_^**T**^
4.2 Optimal rotation matrix, **R**_3×3_ = **USV**^**T**^
4.3 The final optimal rotation matrix, **R**_**A**_ with (3 + *k*) × (3 + *k*) dimension:
4.4 Rotate the second curve with respect to first curve, i.e., ${\mathbf{Q}}_{{{\mathbf{2R}}}} {\mathbf{ = Q}}_{{\mathbf{2}}} {\mathbf{R}}_{{\mathbf{A}}}$

#### Step 5

Achieve optimal matching by dynamic programming as follows

5.1 At first, compute the weights of all edges,
  EW (r, s) = edge weight calculation between vertex for r = 1 to n vertices of **Q**_**1**_ and s = 1 to n vertices of ${\mathbf{Q}}_{{{\mathbf{2R}}}}$
5.2 Find out the shortest path using Floyd–Warshall all-pairs shortest-path algorithm and matching of edge weights
5.3 Obtain **G** (gamma function values), **T**_**g**_ (gamma change point parameter values) and the minimum distance (squared L^2^ distance between matched curves)
5.4 Obtained second curve $({\mathbf{Q}}_{{2{\text{R}}}}^{ *} )$ after optimal re-parameterization.

#### Step 6

The same procedure as given in step 3 is used to calculate a new change point parameter **T**_**r**_ and the corresponding SRVFs, **Q**_**1r**_ and **Q**_**2r**_ are recalculated. Finally, obtained geodesic distance [θ = cos−1(d)] between the curves, where ${\text{d}}= ({\mathbf{Q}}_{{{\mathbf{1r}}}} .{\mathbf{Q}}_{{{\mathbf{2r}}}} ){\mathbf{T}}_{{\mathbf{r}}}^{{\mathbf{T}}}$. The symbol ‘.’ represents the dot product of the matrices.

### b. Evaluation criteria

The proposed algorithm has been implemented in R software. In order to evaluate the performance of the proposed algorithm for protein 3D structure comparison with existing algorithms i.e., (1) CE, (2) jFATCAT and (3) ESA, the benchmark data was collected from the literature [23]. Further, distance matrices based on all four 3D structure comparison algorithms mentioned above have been obtained for the benchmark data. The performance of the 3D structure protein comparison algorithms can be evaluated through cluster analysis using distance matrices. Different statistical performance measures such as rand index, precision, recall and F-measure were used for this evaluation.

### R package development

**Fig. 2:**
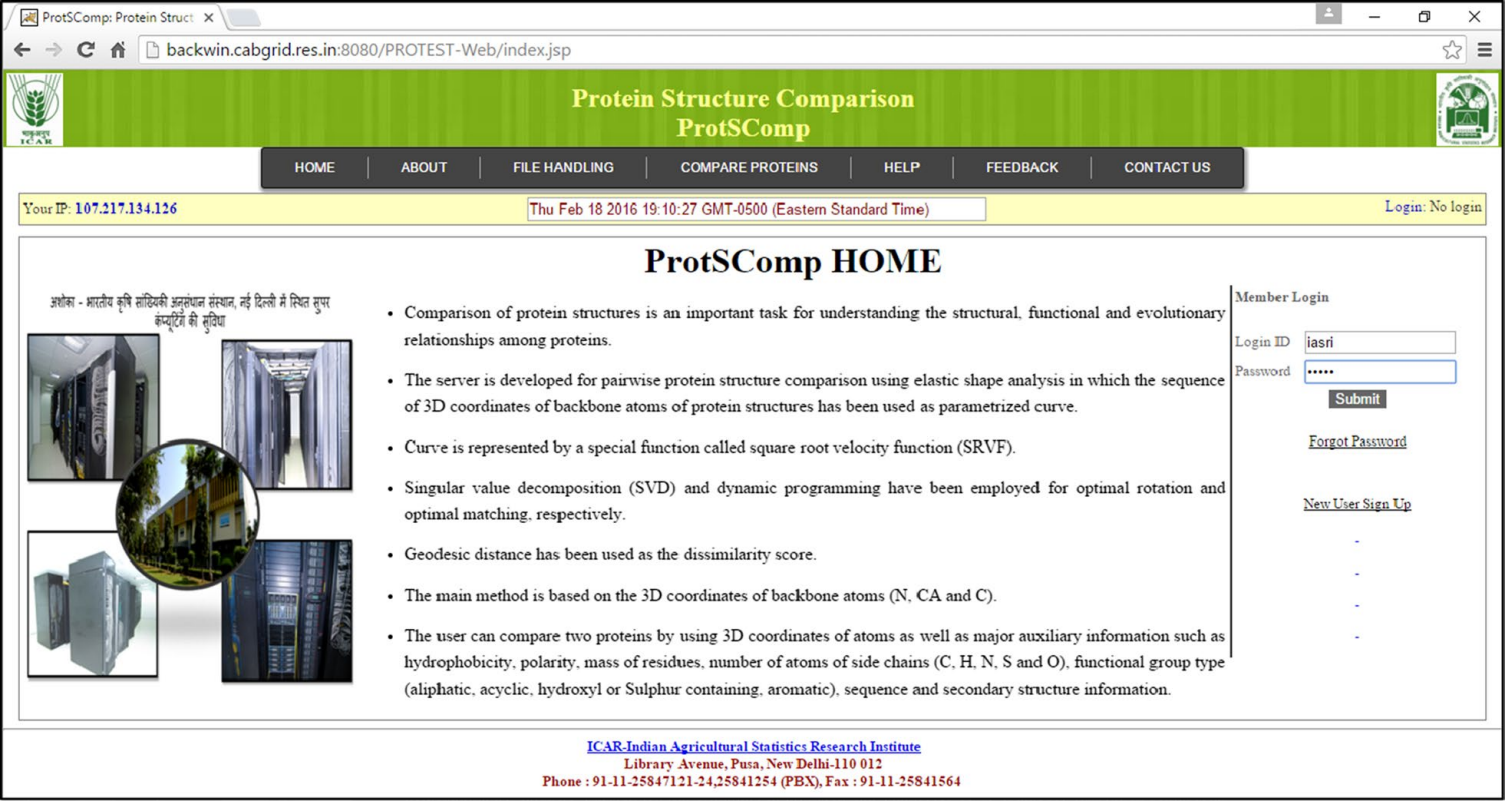
Home page of ProtSComp after user has logged in

The proposed algorithm for comparing protein 3D structures has been developed as an R package [41]. R packages, viz., Bio3D, Rpdb and rgl have been used in downloading PDB files, reading the PDB files and visualization respectively [42–44]. Further, based on this developed R package, a web based server ProtSComp has been implemented (Fig. 2). The server is accessible from http://www.backwin.cabgrid.res.in:8080/ProtSComp. In this web server, R package serves in back-end execution, Java Server Pages (JSP) as server side scripting language, and Cascading Style Sheets (CSS), HTML and Javascript as client side programming language.

### Benchmark data

Two datasets of protein structures from structural classification of proteins (SCOP) [6, 7] database have been taken as benchmark datasets. These datasets were also used by Liu et al. [23] for evaluation of algorithms for 3D structure comparison of proteins. First dataset comprises of 50 proteins from five important SCOP classes with 10 proteins from each class, i.e., class I [All α proteins], class II [All β proteins], class III [α and β proteins (α/β)], class IV [α and β proteins (α + β)] and class V [Multi-domain proteins]. Second dataset consists of 100 proteins structures from three important classes, having 45 proteins from class I, 40 from class II and 15 from class III of SCOP database.

### Computation of distance matrix

The distance matrix of size N×N for N protein structures were computed for all four algorithms i.e.,, (1) CE, (2) jFATCAT, (3) ESA and (4) proposed algorithm, The distance matrices for first three existing algorithms are based on 3D coordinates of backbone atoms, however the proposed method also incorporates auxiliary information along with these 3D coordinates. In order to make this distance matrix uniform, a sigmoid function has been used for conversion of values of geodesic distance and RMSD to common similarity measures between 0 and 1.

### Performance measures

In order to compare the proposed algorithm with commonly used existing algorithms for 3D protein structures, number of clustering techniques such as K-Means, C-Means, Spectral K-Means clustering techniques have been used. It is noted that the results of clustering is not unique as it depends on clustering algorithms used for the analysis. In case of large datasets having known number of classes, the non-hierarchical clustering performs better than the hierarchical clustering. Therefore, the above clustering techniques are likely to perform best in a given situation. The performance of these algorithms for each of these clustering techniques was evaluated based on rand index (RI), recall, precision and F-measure. These evaluation measures have been calculated based on confusion matrix (Table 1). The performance measure for each of the algorithms for a given clustering technique is an indicative measure to evaluate the performance of the respective algorithm, as the clustering is applied on the distance metric generated from the corresponding algorithm.

**Table 1:** Confusion matrix

Group	Predicted class 1	Predicted class 2	…	Predicted class i	…	Predicted class n
True class 1	M_11_	M_12_	…	M_1i_	…	M_1n_
True class 2	M_21_	M_22_	…	M_2i_	…	M_2n_
:	:	:	…	:	…	:
True class i	M_i1_	M_i2_	…	M_ii_	…	M_in_
:	:	:	…	:	…	:
True class n	M_n1_	M_n2_	…	M_ni_	…	M_nn_

In Table 1, *M*_*ii*_ where *i* = *j* is the number of true positives for ith class, i.e., pair of proteins that are classified correctly as per the SCOP database classes; *M*_*ji*_ where $i \ne j$ is the number of false positives, i.e., pair of proteins that are classified incorrectly as correctly identified but rejected. *M*_*ij*_ where $i \ne j$ is the number of false negatives, i.e., pair of proteins that are classified incorrectly as incorrectly identified but accepted; *M*_*ij*_ where *i* = *j* is the number of true negatives for ith class, i.e., pair of proteins that are classified correctly as incorrect identified and also rejected. Based on these values, RI, recall, precision and f-measure are calculated as follows

$$RI_{i} = \frac{{\mathop \sum \nolimits_{i} M_{ii} }}{{\mathop \sum \nolimits_{ij} M_{ji} }} \quad where\;j \ne i ,$$

$$Precision_{i} = \frac{{M_{ii} }}{{\mathop \sum \nolimits_{j} M_{ji} }}\quad where\;j \ne i,$$

$$Recall_{i} = \frac{{M_{ii} }}{{\mathop \sum \nolimits_{j} M_{ij} }} \quad where\;j \ne i$$

$$F - Measure = \frac{2*(Precision*Recall)}{(Precision + Recall)}$$

## Results and discussion

In earlier study for comparing two protein structures based on ESA using only backbone atoms resulted with classification accuracy of 80.73 and 92.10 % for the first and second dataset of proteins respectively [23]. The proposed algorithm is based on ESA using either centroid of backbone atoms (ESA-MC-BB) or C_α_ (ESA-CA) along with dihedral angles as geometric property of molecular structure. Further, in order to improve the alignment molecular auxiliary information such as hydrophobicity (ESA-MC-BB + HP or ESA-CA + HP), polarity (ESA-MC-BB + POL or ESA-CA + POL), mass of residues, functional group type and number of side-chain atoms along with back bone atoms have been considered. In order to compare the effect of auxiliary information on classification accuracy and computational time, different combinations of molecular auxiliary information has been included through the proposed algorithm and analysis was done on the first and second datasets using different clustering techniques. It was observed that either the classification accuracy has increased or there is substantial reduction in computational time of comparison of two protein structures through proposed algorithm. The performance measures such as RI, precision, recall and f-measure are shown in Table 2.

**Table 2:** Performance measures of 100 proteins dataset from ESA, CE and jFATCAT methods at class level with computational time

Method/levels	Time (hours) for N×N comparison	Measure	Spectral K-means	K-means	Fuzzy C-means
CE	126.18	Precision	0.9600	0.8622	0.7141
		Recall	0.9333	0.7573	0.9792
		F-measure	0.9465	0.8064	0.8259
		RI	0.9694	0.9538	0.9226
jFACTCAT	019.14	Precision	0.6653	0.4929	0.5058
		Recall	0.6043	0.5019	0.6741
		F measure	0.6333	0.4974	0.5780
		RI	0.8554	0.8430	0.8154
Original ESA	020.40	Precision	0.8396	0.5075	0.4812
		Recall	0.7563	0.7744	0.6347
		F measure	0.7957	0.6132	0.5474
		RI	0.9420	0.8248	0.8032
ESA-MC-BB	002.20	Precision	0.7767	0.5523	0.5710
		Recall	0.9275	0.6277	0.5232
		F measure	0.8454	0.5876	0.5461
		RI	0.9359	0.8440	0.8338
ESA-MC-BB + HP	002.20	Precision	0.9168	0.5058	0.5699
		Recall	0.8400	0.7925	0.5307
		F measure	0.8767	0.6175	0.5496
		RI	0.9557	0.8298	0.8369
ESA-MC-BB + POL	002.20	Precision	0.8974	0.5416	0.5576
		Recall	0.8165	0.6000	0.5088
		F measure	0.8551	0.5693	0.5321
		RI	0.9444	0.8159	0.8322
ESA-CA	002.20	Precision	0.8572	0.5075	0.5322
		Recall	0.7621	0.7744	0.4800
		F measure	0.8069	0.6132	0.5048
		RI	0.9364	0.8961	0.8234
ESA-CA + HP	002.20	Precision	0.8495	0.7588	0.5576
		Recall	0.7525	0.6997	0.5088
		F measure	0.7981	0.7281	0.5321
		RI	0.9411	0.9020	0.8322
ESA-CA + POL	002.20	Precision	0.8572	0.5058	0.5205
		Recall	0.7621	0.7925	0.4672
		F measure	0.8069	0.6175	0.4924
		RI	0.9297	0.8388	0.8194

The proposed algorithm was evaluated with existing algorithms based on computational time (Table 2). It is observed from the table that the computational time required for comparison of 100 proteins dataset for CE, jFATCAT and Original ESA are more i.e., 126.18, 19.14, 20.40 h respectively. However, our proposed algorithm takes considerably less time i.e., 2.20 h. Therefore, our algorithm is quite efficient in terms of computational time.

It has been reported earlier [23] that original ESA, which is based on all backbone atoms of the protein structures, time consumed to perform the experiment of 100 protein structures comparison was recorded on a desktop computer (8 GB RAM; 64-bit Windows 7 OS; MATLAB version 7.9.0) was 59 h but when it is implemented in R, it took 20.40 h. Under the same setup, the proposed algorithm implemented using R (version 3.1.3), the computing time varied from 2.80 to 3.00 h. As per algorithm, we employed three different criterion to evaluate variation in the results based on various geometric properties such as (1) backbone atoms, (2) c-alpha and (3) centroid of backbone atoms along with orientation (dihedral angles). In addition to this, the auxiliary information i.e., hydrophobicity and polarity for each amino acid in a protein are considered. In view of time complexity, the earlier ESA method used 3D coordinates of all backbone atoms (N, Cα and C) [23]. In this case, if there are n number of amino acids (or residues) then the length of curve will be 3*n* as each amino acid is being represented by three atoms. The rest of the criterion i.e., (2) and (3) are based on n number of centroid 3D coordinates, five molecular properties and three geometric properties as dihedral angles. These dihedral angles are phi, psi and omega for each amino acid, and five molecular properties as mentioned above. Hence, the proposed algorithm is faster than the existing ESA [23] as the proposed algorithm is based on n number of data and earlier ESA is 3n in data size that reduces the one-third of the computational time without much compromising on performance.

The performance of Spectral K-Means clustering is better for comparison of various algorithms in terms of precision followed by Fuzzy C-Means clustering. However, results obtained by K-Means clustering techniques are not satisfactorily in terms of precision.

The performance of CE in terms of recall, F-measure and RI is much better in comparison to all existing methods i.e. jFATCAT, original ESA etc. However, in case of proposed algorithm (ESA-MC-BB), recall and RI are comparable with CE through Spectral K-Mean clustering. It may be noted that computational time for CE is 126.18 h whereas proposed algorithm takes around 2.20 h for same task.

In terms of RI, the accuracy for the first and second set of proteins increased up to 88.72 and 95.57 %, respectively when hydrophobicity was included as auxiliary information. It was also observed that the RI of the protein structures of second set shows 94.11 % accuracy when distance was calculated using 3D coordinates of C_α_ atoms and hydrophobicity as the auxiliary information. This may be due to the fact that the proposed algorithm used only single coordinate for each residue as centroid of backbone atoms or C_α_ with dihedral angles (phi, psi and omega). These dihedral angles are indirectly using all coordinates by single data point with three more additional parameters. The proposed algorithm also included molecular properties of each residue and hence the results of proposed algorithm are comparable with ESA of all backbone atoms.

In another experiment, the computing time of the different methods of protein structure comparison [22], viz., combinatorial extension (CE) [30], Matt [32], MUSTANG [33] and ESA [22, 23] have been recorded for varying number of residues along with the proposed algorithm. The computing time of the existing and proposed algorithm are given in Table 3. In case of 100 residues, MUSTANG required slightly less time as compared to proposed methods. The computational running time of the proposed methods are significantly smaller than the existing algorithm in case of protein containing larger than 100 residues.

**Table 3:** Computational time (in seconds) required in comparing two protein structures using different methods

Method	~100 residues	~200 residues	~300 residues
Matt	1.300	3.000	5.100
MUSTANG	0.160	2.300	2.100
ESA	1.200	2.600	15.000
Proposed method (ESA-MC-BB)	0.740	1.040	1.540
Proposed method (ESA-CA)	0.556	0.745	1.466

The proposed method performed better in terms of classification accuracy due to the inclusion of side chain/amino acid properties. This is due to the fact that inclusion side chain/amino acid properties provides more appropriate representations of protein structures as per elastic shape analysis. Further, hydrophobicity plays important role in the folding of protein structures as the hydrophobic residues tend to moves towards inner structure of the protein whereas, hydrophilic atoms moves towards the protein surface during protein folding [37]. Therefore, incorporation of this feature as auxiliary information led to the improvement in classification of proteins.

### Web server implementation

**Fig. 3:**
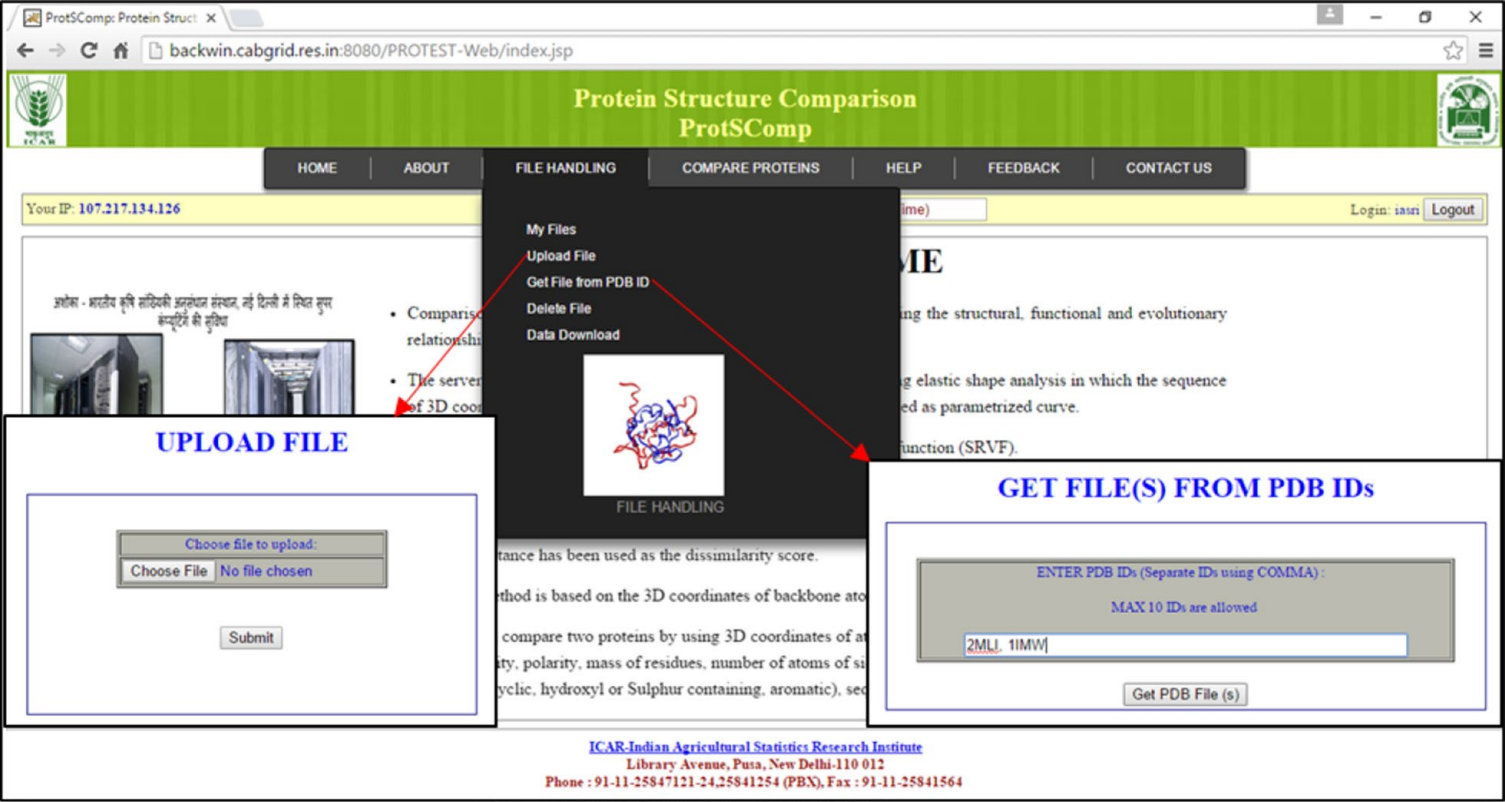
Upload file on ProtSComp server

**Fig. 4:**
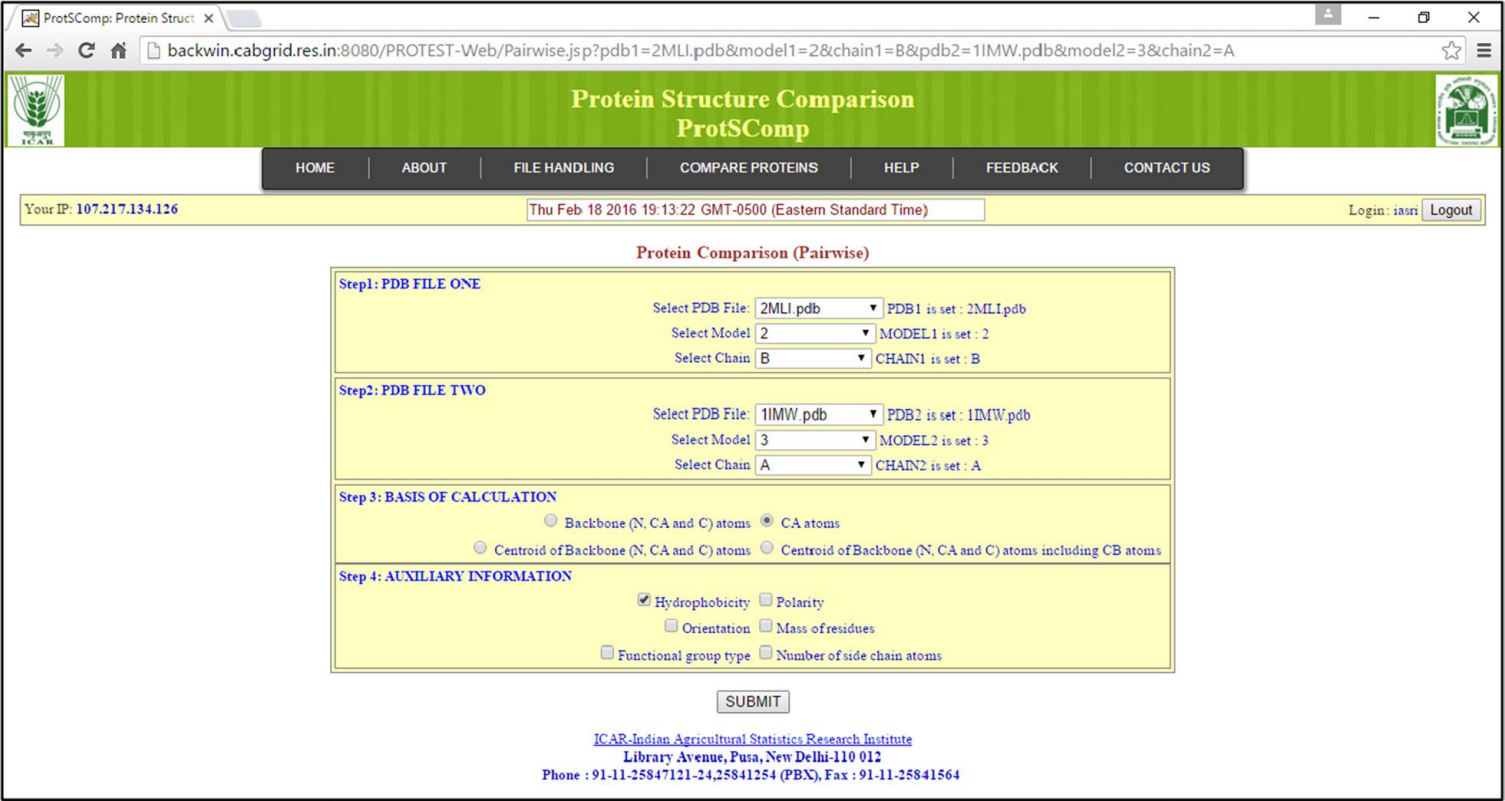
Provision for various parameter selections and options such model, chain and auxiliary information

**Fig. 5:**
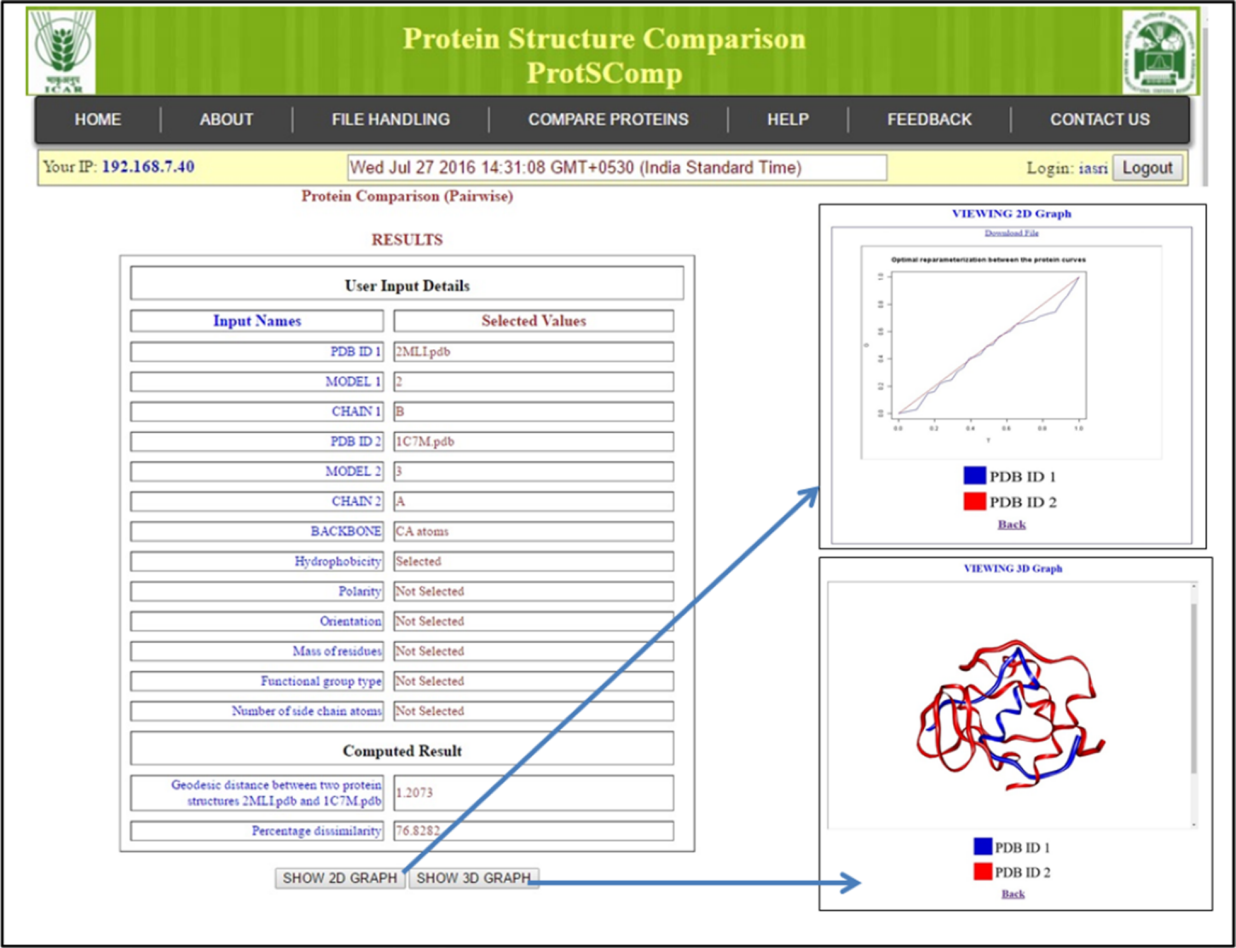
Presentation of final result as geodesic-distance in text (*left*) and graphical (*right*) form

In order to use the proposed algorithm, a web based tool (ProtSComp) has been developed. In this tool, two proteins can be compared. The number of residues for the comparison is based on selection of model and chain. The user can upload PDB file(s) or give the PDB ID(s) (Fig. 3), select a model, a chain for each protein under consideration (Fig. 4). Protein structures can be compared using different criteria based on geometric and auxiliary information as discussed above (Fig. 4). As an example, for comparing two protein structures with PDB Ids i.e. “2MLI.pdb” (Model 2 and Chain B) and “1IMW.pdb” (Model 3 and Chain A) have been illustrated (Fig. 4). Finally, result outputs can be seen in terms of geodesic distance along with selected optional criterion, model and chain for both proteins. Also, optimal matching superimposed structure of both can be visualized in separate window (Fig. 5).

## Conclusions

With the advent of high-throughput methods, the availability of structural information of proteins is increasing at a much accelerated pace. There is a requirement of automatic annotation and classification of proteins in order to save resources in terms of time. Therefore, the fast and efficient algorithm is developed that will find the best alignment between two protein structures.

In this study, a computationally efficient algorithm has been developed in terms of run time for comparing protein structures based on ESA approach. The 3D coordinates of protein backbone atoms using different criteria have been used including the auxiliary information based on side-chain properties residue wise. The proposed algorithm has been developed using R.

The proposed algorithm performed equally well in terms of accuracy with respect to existing techniques due to the inclusion of side chain and amino acid properties. Inclusion of hydrophobicity as auxiliary information shows better result since it plays important role in the folding of protein structures. Incorporation of molecular properties as auxiliary information led to the improvement in comparison of two protein 3D structures. The proposed algorithm is faster in terms of computational time than the existing algorithm since it is based on n number of data instead of 3n in data size employed by existing algorithms.

## Abbreviations

RMSD: root mean square deviation
DALI: Distance mAtrix aLIgnment
SRVF: square root velocity function
SVD: singular value decomposition
GUI: graphical user interface
UPGMA: unweighted pair group method with arithmetic mean
RI: rand index
CE: combinatorial extension
ESA: elastic shape analysis
SCOP: structural classification of proteins
